# Mitochondrial ROS versus ER ROS: Which Comes First in Myocardial Calcium Dysregulation?

**DOI:** 10.3389/fcvm.2016.00036

**Published:** 2016-10-10

**Authors:** Ruchi Chaube, Geoff H. Werstuck

**Affiliations:** ^1^Thrombosis and Atherosclerosis Research Institute, McMaster University, Hamilton, ON, Canada

**Keywords:** heart failure, endoplasmic reticulum stress, mitochondrial ROS, calcium dysregulation, unfolded protein response

Cardiomyocyte excitation–contraction coupling is tightly regulated through coordinated calcium release and uptake that is facilitated by two proteins in the sarco/endoplasmic reticulum (SR/ER) membrane, the ryanodine receptor (Ryr), and the sarco/endoplasmic reticulum Ca^2+^-ATPase (SERCA), respectively. Dysregulated calcium handling is a hallmark in cardiac dysfunction (1–3). Our understanding of the molecular mechanisms that underlie these calcium handling anomalies and their precise role in the associated pathologies remains incomplete. A growing body of indirect evidence has implicated a role for redox signaling, and the mitochondria have been thought to be the primary source of reactive oxygen species (ROS) (4–10). Research in this area suggests that ROS generated from the mitochondria alters the redox milieu around the SR/ER–mitochondria interface causing excessive oxidation of the cysteine residues in Ryr and SERCA that results in an increase in calcium release and decrease in uptake, respectively, thus depleting the SR/ER Ca^2+^ stores and causing dysregulated downstream signaling (4–10). However, many questions remain unanswered, and the source of the ROS has not been directly validated.

Mitochondria and SR/ER are in close apposition and the interface, commonly known as the mitochondrial-associated ER membrane (MAM), is believed to act as the focal point for this signaling. Our knowledge of calcium signaling in cardiac pathologies, where ER and oxidative stresses are predominant (11–14), suggests that calcium may, in fact, be the cause, rather than the effect, of mitochondrial ROS. This implies that calcium overload signals mitochondria to produce lethal levels of ROS. Therefore, do alternative sources of ROS initiate the redox imbalance that causes calcium dysregulation? In this article, we present data that suggest that, in addition to mitochondrial ROS, ROS originating in the ER may indeed explain gaps existent in the field cardiovascular pathophysiology.

## ER ROS and Calcium Signaling at the MAMs

The ER is a primary site of protein synthesis and posttranslational processing. The concomitant production of hydrogen peroxide (H_2_O_2_), as a byproduct of the Ero1α-PDI protein folding pathway and a high glutathione disulfide:glutathione (GSSG:GSH) ratio keeps the SR/ER in a slightly oxidative state (15). The redox state of the ER is critical for the folding process and factors that lead to the accumulation of the oxidizing equivalents in the ER give rise to mis/unfolded polypeptides, a condition known as ER stress. ER stress initiates the activation of the unfolded protein response (UPR). The UPR increases the capacity of the protein folding machinery resulting in the production of more oxidative equivalents, and further deteriorating the redox state. The cell handles the exacerbated stress by inhibiting protein synthesis and activating the ER-associated degradation (ERAD) pathway to remove the terminally misfolded proteins (16). An important feature of the stressed condition is the release of calcium from the ER by the opening of calcium channels, including the ryanodine receptor and inositol-3-phosphate receptor (IP3R) (17).

Regulated calcium release from the SR/ER is essential for several cellular processes including muscle contraction. Intra SR/ER calcium also plays an important role in ER chaperone function in protein folding. To maintain calcium homeostasis calcium is returned to the SR/ER through SERCA. During pathophysiological conditions, both the release and uptake of calcium from the ER are dysregulated, resulting in enhanced calcium release (18, 19). Much of the released calcium is taken up by the mitochondria. Mitochondrial calcium overload can lead to mitochondrial dysfunction and the initiation of a cascade of pro-apoptotic events (20). The checkpoint for this phenomena lies on the MAMs and the calcium handling proteins, Ryr (excitable cells) (21) and IP3R (non-excitable cells) (20) on the ER, and VDAC on the outer mitochondrial membrane (20) have been shown to reside at this interface where they function to mediate in the facile transfer of calcium from the SR/ER to mitochondria. Calcium within the mitochondria generates superoxide that is presumed to be the marker for oxidative stress (22). This led to the design of mitochondrial targeted antioxidant therapies; however, these therapies have largely failed in the clinical trials relating to cardiovascular disorders (23, 24).

## Physiological Production of Mitochondrial and ER ROS

Mitochondria are traditionally believed to be an important source of ROS, and the role of mitochondrial ROS in mediating cardiac pathophysiology has been shown in numerous studies (25, 26). However, in the context of the myocardium, it is not clear that mitochondrial ROS actually cause calcium dysregulation in the SR/ER. The proximal mitochondrial ROS, superoxide $\left({\text{O}}_{2}^{-}\right)$ is produced when the electrons leak while passing through the electron donors in the complex I and complex III of the mitochondrial respiratory chain. Superoxide, being unstable and short lived, is catalytically dismutated into H_2_O_2_ by the manganese superoxide dismutase (MnSODs) in the mitochondrial matrix; this form can readily move across the biological membranes to oxidize various targets. As noted by Murphy (27), two factors primarily determine the rate of ${\text{O}}_{2}^{\cdot -}$ production by mitochondria; the enzyme concentration carrying the electron donor (NADH, NADPH, and CoQH_2_); and the proportion of the electron donor present in the redox form that can react with O_2_ to generate ${\text{O}}_{2}^{-}$. The latter may be governed by the local environment, such as the proton motive force, mitochondrial ATP synthesis, and other parameters including pH, O_2_ concentration, mutation, posttranslational modifications, etc. Similarly, the oxidizing power of H_2_O_2_ is kept low by the action of the cellular enzymes. Besides catalase, which was the first enzyme to be discovered that scavenges H_2_O_2_ and is not abundant in mitochondria (28, 29), another enzyme system called the thioredoxin (Trx) system has gained importance with respect to the scavenging of mitochondrial H_2_O_2_. The Trx system consists of Trx, NADPH-dependent Trx reductase, and peroxiredoxins (Prx), and in an electron relay, the Trx reductase transfers electrons from NADPH to Trx which then transfers them to Prx to convert H_2_O_2_ into H_2_O (30–32). Most importantly, Trx2 has been shown to buffer mitochondrial H_2_O_2_ in cardiomyocytes (33). Presumably, under physiological conditions, there is minimal ROS production by mitochondria, but this may be enhanced by pathophysiological conditions such as myocardial ATP depletion and high proton motive force that results in decreased muscle contractility and may leave the electron donor in a redox form that favors ROS generation. A recent study by Santulli et al. (10) demonstrated that in heart failure, the mitochondrial calcium overload mediated through the leaky Ryr2 increases the ROS production in mitochondria which subsequently oxidizes the Ryr2, thereby enhancing the SR/ER Ca^2+^ leak. This viscous cycle of Ca^2+^ leakage, calcium overload, and ROS generation completely paralyzes cardiac contractility. Noteworthy, the signal for such phenomena originates upstream when calcium dysregulation has already begun from the SR/ER, suggesting that mitochondrial ROS are merely a result of impaired calcium release under these scenarios. Moreover, ample evidence indicates that mitochondrial ROS have a far greater role in bringing oxidative damage to the cell, and in most pathologies, such as neurodegenation, aging, and diabetes (34–36), this initiates apoptosis. An interesting observation made by Leadsham et al. (37) demonstrated that increased ROS levels following mitochondrial dysfunction are mainly due to the ${\text{O}}_{2}^{\cdot -}$ production from the NADPH oxidase, Yno1p on the ER surface in yeast. While this study lacks mechanistic details (38), it does suggest that mitochondria may not be the lead source of ROS even when it is dysfunctional.

On the other hand, considering ER ROS, H_2_O_2_ is a prerequisite for oxidative folding and is a regular and direct byproduct of the process. Evidence shows that the H_2_O_2_ produced by the PDI-Ero1α pathway is locally detoxified by another enzyme – PrxIV. H_2_O_2_ oxidizes the two cysteine residues in PrxIV, after which PrxIV oxidizes the PDI_(red)_ during the oxidative folding of proteins. It has been postulated that PrxIV helps to maintain the redox balance in the ER lumen by preventing H_2_O_2_ accumulation. However, under conditions of UPR activation, excessive H_2_O_2_ may hyperoxidize PrxIV, inhibiting its activity, thereby disturbing the redox balance (39). Consistent with this observation, it is intuitive that H_2_O_2_ can also oxidize other proteins in the ER.

## Redox Regulation of Calcium Handling Proteins

Interestingly, ER stress signals result in enhanced calcium release and inhibition of Ca^2+^ uptake into the ER. SERCA, IP3R, and Ryr channels are all subjected to redox regulation. The activity of SERCA is regulated by S-glutathionylation, and evidence shows that SERCA is inhibited by the ROS-mediated S-oxidation of the conserved Cys 674 (40). Likewise, ROS alters the binding of inositol 1,4,5-trisphosphate (IP3) to the IP3R and affect its activity (41). For Ryr calcium channels, multiple cysteine residues are redox regulated. Sun et al. (42) identified the nature of 93/100 cysteine residues in the skeletal muscle isoform of ryanodine receptor (Ryr1). The redox-regulated Cys residues were categorized by their dependency toward muscle oxygen tension. It was found that 13 Cys residues are subjected to pO_2_-dependent S-oxidation. Furthermore, eight Cys residues were found to be oxidized at high versus low pO_2_ when NADPH was supplemented to enhance NADPH oxidase 4 (NOX4) activity. Importantly, these redox-regulated Cys residues were shown to localize to the binding regions of two interacting partners of Ryr1 – FKBP12 and calmodulin, which, when bound to Ryr favors a closed channel configuration. In a separate study, Sun et al. (43) demonstrated that NOX4 colocalizes with Ryr1 on the SR/ER membrane and directly generates H_2_O_2_ that is responsible for oxidizing the set of redox regulatory Cys residues in Ryr1. This evidence adds NOX4 as a yet another direct source of H_2_O_2_ in the SR/ER. The cardiac Ryr (Ryr2) contains 89 Cys residues, it has been shown that hyperoxidation of the channel increases its open probability, which makes the channel leaky (44), a pathophysiological condition predominant in heart failure. However, the identities of the redox-regulated Cys residues are yet to be determined. Another enzyme, calmodulin-dependent protein kinase II (CaMKII), has been shown to play a preeminent role in regulating the activity of both Ryr2 and SERCA. CaMKII inhibits phospholamban by phosphorylating it at Thr17, which activates SERCA and increases Ca^2+^ uptake into SR/ER (45). Similarly, CaMKII phosphorylates Ryr2 at Ser2814 and possibly at Ser2808 and induces both diastolic SR Ca^2+^ leak and sensitizes Ryr2 to Ca^2+^-induced Ca^2+^release during excitation–contraction coupling (45). Interestingly, oxidation of CaMKII has been linked to heart failure. It has been shown that H_2_O_2_-mediated oxidation of pair of methionine residues (Met281/282) in CaMKII activates the kinase activity and phosphorylates Ryr2, rendering it leaky and thereby depleting the SR Ca^2+^ content. However, methionine oxidation of CaMKII results from oxidative stress generated from mitochondrial H_2_O_2_ and subjects’ cardiomyocytes to apoptosis during heart failure (46).

## Conclusion

**Figure 1:**
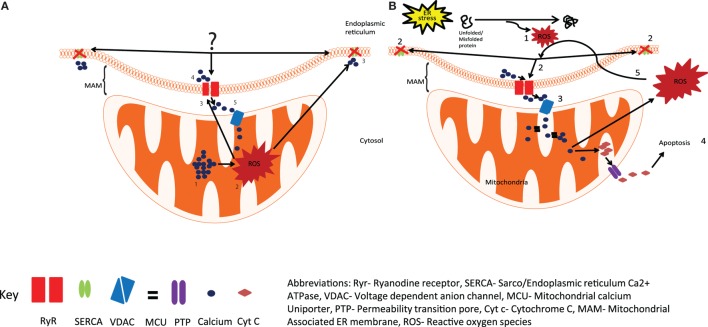
**Production of reactive oxygen species (ROS) by the ER and mitochondria**. **(A)** The current view is that the calcium overload in mitochondria (1), generates ROS by activating Kreb cycle (2), leading to the oxidation of Ryr and SERCA (3), resulting in increased calcium release through Ryr and reduced calcium uptake from SERCA (4), the released calcium is taken up by mitochondria (5), resulting in increased ROS production. **(B)** We hypothesize that activation of UPR in response to ER stress results in the generation of ROS (1) and leads to the oxidation and dysregulation of Ryr and SERCA (2), enhanced calcium release from the ER leads to mitochondrial calcium uptake (3), resulting in enhanced mitochondrial ROS production and the release of Cyt *c* into the cytosol, and the initiation of apoptosis (4), excessive mitochondrial ROS can further exacerbate impaired calcium signaling through Ryr and SERCA (5). Abbreviations: Ryr, ryanodine receptor; SERCA, sarco/endopiasmic reticulum Ca^2+^ ATPase; VDAC, voltage-dependent anion channel; MCO, mitochondrial calcium uniporter; PTP, permeability transition pore; Cyt *c*, cytochrome *c*; MAM, mitochondrial-associated ER membrane; ROS, reactive oxygen species.

The potential role of the ER in the induction of calcium dysregulation adds a new dimension to the already established role of ROS from the mitochondria. It is plausible that the skewed production of ROS can begin well before the mitochondrial mediation during the adaptive phase of UPR when the protein folding capacity is enhanced that alters the redox states of Ryr and SERCA and triggers an aberrant calcium release and uptake, respectively. Moreover, the ROS thus generated would better serve both the temporal and spatial aspects in oxidizing the Cys residues of RyR and SERCA (Figure 1). Future research will directly test the role of redox signaling initiating from the SR/ER; this could lead to the identification of novel upstream targets for the development of new, more efficient therapies.

## Author Contributions

RC wrote the manuscript and GHW critically revised the manuscript.

## Conflict of Interest Statement

The authors declare that the research was conducted in the absence of any commercial or financial relationships that could be construed as a potential conflict of interest.
